# Supplementary material for: Understanding Nutrition and Metabolism of Threatened, Data-Poor Rheophilic Fishes in Context of Riverine Stocking Success- Barbel as a Model for Major European Drainages?
Source: Biology (Basel). 2021 Nov 29;10(12):1245. doi: 10.3390/biology10121245 (PMC8698400; doi:10.3390/biology10121245)
Supplement: Supplementary file 1 [file biology-10-01245-s001.zip › biology-1480749-supplementary.pdf]

SUPPLEMENTARY

MATERIALS AND METHODS

Selection of model species and calculation of target nutrition

Firstly ('Approach A'), the nutrient composition of 'primary' natural food item is multiplied by 0.9. Hence, the formula for 'Approach A' is as follows: target nutrient level= crude nutrient content in a natural food item × 0.90. The justification of multiplication factor 0.9 is that primary food item might not occupy 100% of the food share in the gut, even when its supply is bountiful; a minor food share in the gut (~10% or 0.1) may still be contributed by some filler food items and/or non-nutritional ballast (e.g., detritus, sand) which may not be the key driver of growth (supplementary appendix-I). For approach A, some additional resources are required for calculation. Like the crude nutrient composition of some everyday food items available in the habitat (supplementary appendix-II). For our calculation purposes, we have used 'chironomid larvae' (supplementary appendix-II and footnotes in Table 2) as a proxy of primary natural food items for barbels. Approach A can be limited by the fact that quite often, preferred natural food item in the habitat may become unavailable. Under such circumstances, secondary and tertiary food items with most likely lower crude nutrient content would contribute more potentially undermining the estimates (supplementary appendix-I). The proximate composition of such food items (supplementary appendix-II), when used for calculation, should be dealt with carefully.

Secondly ('Approach B'), the optimum dietary nutrient levels can be estimated by dividing body nutrient content (live matter basis) with the average nutrient retention of cyprinids. The formula for 'Approach B' is as follows: target nutrient level= [body nutrient × (100 ∕ retention)]. For Approach-B, the analyzed body composition data is needed for calculation (Table 1). Besides, a typical cyprinid retention scheme needs to be assumed [1, 2]. For our calculation purposes, we have used an average cyprinid retention (% of crude intake) scheme with protein retention 31%, energy (lipid) retention 38%, and phosphorus retention 45% (adopted from Roy et al. 2021 communicated). Protein and amino acids digestibility [3, 4] and retention (Roy *et al.* 2021 communicated) resemble each other, so the values for protein can be grossly used for amino acids too [2]. However, the same cannot be said for fatty acids [2]. Fishes, in general, are known to manipulate their body fatty acids stoichiometry *de-novo* to some extent [5, 6]. So, the final content in the whole body was used as it is. The approach B may also give under-estimations if fishes are of poor physiological status from the wild are used for calculation. To be unbiased by food availability and living conditions, it is advised to sample several fishes during the peak feeding period of the vegetative season (usually in summer) or in the middle of the vegetative season.

Lastly ('Approach C'), the[7] protein recommendations for *Cyprinus carpio* common carp below 20 g body size, to match the life stage of juvenile rheophilic cyprinids meant for hatchery rearing, was considered. Since [7] recommends essential amino acids requirements for common carp that were given against an optimum protein requirement of 32% (which is meant for 100-300 g body sizes), we re-calculated the amino acids based on their % contribution to optimum protein level and re-adjusted them to 45% protein which is optimum for below 20 g body sizes. Optimum phosphorus requirement (1.2%) was taken from [2]. Rest of the values (lipid, fatty acids) were used as it is.

In terms of commercial diet selection from a spectrum of premium products available in the market, be aware that the actual protein and lipid content could vary by average −5% and −17% (respectively) than promised on the labels, *i.e.,* lower than declared, at times (author observations). Therefore, such interferences might be factored in the calculations; if any potential diet falls 'just' on the target margin (range).

Selection of experimental diets and feed preparation

Commercial feed(s) were first grounded to a fine powder in an electric grinder (River Systems SRL, Mistral 50 L, Italy), mixed with 30% water (300 ml per kg powder) in an electric mixer (Fac srl, Cutter LT 3, Italy) to prepare a dough. The dough was introduced into a cold pelletizer (Bottene, Pasta Machines dal 1805, Italy) fitted with a rotating electric slicer (AUTOM Milano, Italy). Cold extruded noodles were sliced into cylindrical pellets (5–7 mm long, 1.5–2 mm diameter). The pellets were fan-dried at room temperature for 8 hours, following which they were loaded onto a hot air oven (BMT, INCUCELL LSIK-B2V, Czech Republic) for 48 hours at 45°C with intermittent turning and releasing of water vapors. The dried pellets were cooled down at room temperature for 30 minutes, dust and fines were separated, and they were stored in the refrigerator (in dry mode) until further use.

Table S1: Additional information about the selected diets (see also, main text Table 2A, B).

Parameters	Diet A *	Diet B *	Diet C*	Diet D*
Non-protein energy to protein ratio (cal mg^{-1})	3.5	3.8	3.3	6.3
Phosphorus to protein ratio (PPR; mg P g $protein^{-1}$)	13.4	9.8	10.0	10.4
Declared ingredients (composition protected)	Fish meal; Wheat gluten; Wheat; Krill meal[#]	Fish meal; Wheat gluten; Soya protein concentrate[#]; Wheat, Wheat starch	Fish meal; Wheat; Processed animal proteins from Poultry[#]; Soya meal feed; Wheat gluten; Corn gluten feed	Corn condensed distillers solubles[#], Soy meal feed; Wheat; Faba bean; Fish meal; Corn gluten feed

*Comparison with label information: analyzed crude protein (**−5%**) and crude lipid (**−17%**) fell short than specified (!). Total P was **+2.4%** excess than specified.

Diet A= whitefish/ trout (expensive) starter (~4 € kg^{-1}) ; **Diet B**= catfish/ trout (less expensive) starter (~2.8 € kg^{-1}); **Diet C**= trout/ catfish grower (~1.45 € kg^{-1}); **Diet D**= carp grower (~1.29 € kg^{-1}).

[#]Novelty in the commercial aquafeed industry (in the recent decade).

Evaluation of growth, factors affecting growth, and physiological performance

Evaluation of experimental results

Data on total body length (TL, cm), body weight (BW, g), duration of culture (days), mean water temperature (°C; trial 1= mean 21.7°C; trial 2= mean 22.8°C), whole body proximate composition (% nutrient of dry matter basis; or, kcal 100 g dry matter^{-1}), total feed and nutrient fed (g per harvested biomass) were used for calculation of following parameters: (a) thermal growth coefficient, TGC (units)= 100× (final weight$^{1/3}$ – initial weight$^{1/3}$) ⁄ sum degree days (°C) [8]; (b) length increment, LI (mm day^{-1})= increment in length ⁄ number of days; (c) Fulton's condition factor, CF = (length, cm× 100) ⁄ (weight, g)^3 [9]; (d) yield (kg m^{-3} day^{-1})= increment in biomass (kg) ⁄ volume (m^3) ⁄ duration of culture (days); (e) nutrient or energy retention (% of intake)= [(carcass nutrient content at end of experiment) – (carcass nutrient content at start of experiment)] ⁄ nutrient intake during experiment [8]; (f) nutrient or energy loss (% of intake)= 100 – retention % [8]; (g) final body size (total length)-at-age.

Additionally, the nutritional traits of the diets were estimated by protein-sparing potential (PSP) and phosphorus-to-protein ratio (PPR). PSP is digestible non-protein energy (NPE): protein ratio in a food item (NPE: P), divided by maximum NPE:P of wild barbel body (~2.38 cal NPE mg protein^{-1}). The closer the value is to 1, the better. Too low or too high PSP is bad (Roy et al., 2021 communicated). PPR is digestible phosphorus to protein ratio (intake) through diet, whose value if >14, may be stressful for the evolutionarily primitive fish kidney (*sensu* [10, 11]; Roy et al. 2021 communicated). For calculating the digestible protein, phosphorus, and energy, representative values of cyprinid gastrointestinal capacity (protein digestibility ~89%, phosphorus digestibility ~37%, energy digestibility 76%) were used from [2]. Physiological performance, on the experimental diets, were assessed by few markers: (a) protein efficiency ratio, *i.e.,* marker of efficient growth (PER, higher the better; [8]; (b) P: N retention ratio, *i.e.,* marker of efficient connective/ skeletal tissue growth in tandem with growing musculature (values near the maximum observed P: N ratio of whole-body wild barbel ~286.5 mg P per g N is ideal; *sensu* [12], and; (c) NPE: gross energy (GE) retention ratio, *i.e.,* a marker of energy metabolism (values near the maximum observed NPE: GE stoichiometry in wild barbel ~0.37 cal NPE reserved per cal GE is ideal; *sensu* Roy et al. 2021 communicated).

All statistical analyses, tests, and graphical modeling were done in RStudio v3.5.1 using default commands or packages like 'ggplot2', 'mgcv', 'e1071', 'dunn.test', 'FSA'. Growth and feed utilization indicators were calculated from 1st quartile, median, mean and 3rd quartile of values of length and/or weight observations of pooled replicates (inter-replicate differences were non-significant, $p>0.05$). The coefficient of variance was used as a measure of body size heterogeneity. The standard deviation of tank biomass, density, and yield were calculated replicate-wise. Statistical tests were selected (parametric= Tukey's HSD test, or non-parametric= Dunn's test with Bonferroni correction) following a Shapiro-Wilk normality test of concerned parameter with alpha level set at 0.05. Semi-upper IR (*i.e.,* median to 3rd quartile) of TGC or LI was benchmarked as 'reasonably good growth.' Detailed descriptions can be found in [2], [13].

At the end of the experiment, following dissection of starved fish, an intact alimentary tract was photographed. The liver was photographed. Gastro-intestinal (belly) fat bodies were visually assessed. Cross-section of the intestinal lumen (rostral part) was photographed, measured for micro-villi height (length), and expert characterization of microvilli structure was done in a third-party veterinary histology laboratory (https://fvl.vfu.cz/cz/oddeleni-histologie). Accidentally, mild autolytic damage did occur in the observations, as starved fish intestine (with active enzymes) was flushed with just distilled water (inserting an Eppendorf micropipette in the lumen) and stored in a mild preservative (Sigma-

Aldrich 4% phosphate-buffered formalin). The steps were probably insufficient in suppressing post-mortem enzymatic autolysis of microvilli. Still, the results are included as part of 'supplementary observations' as there were some patterns in the extent of autolytic damage (diet A and B maximum; diet C moderate; diet D minimum) as inferred in the expert report obtained from the third-party service laboratory. In growth trial 2, signs of any conspicuous gonads were also tracked in early 1+ barbels [14]. All observations in this category were purposively qualitative.

Retrospective evaluation against reviewed metadata

Two separate searches were conducted. Once for wild data and once for captive data. For wild data, keywords used for the search were 'growth', 'length', 'weight', '*Barbus barbus*', 'common barbel', 'river' and/or 'body composition', 'diet', 'food items', 'Europe', 'European' (also in different combinations). For captive data, 'growth trial', 'feeding', 'nutrition', 'artificial diet', 'Barbus barbus' and/or 'captive', 'rearing', 'protein', 'lipid', 'fat', 'energy', 'common barbel', 'reproduction', 'juveniles' were searched (also in different combinations). In the first round, articles were screened either to the end of search page results or up to 120 returned results (e.g., google scholar returning 10 results per page; 20-30 results pages) to pick up relevant articles for building the meta-dataset. From these primary articles, relevant cross-references were identified, and if found missing, they were searched. The screening was also done at this stage if the articles were relevant. As a measure of internal quality check, only peer-reviewed scientific literature (from reputed international or national publishers) was selected at least with English abstract. For the essential grey literature, only items reliably listed in CABI or Agris FAO were selected. Empirical studies, studies on larvae, studies published in predatory journals were not considered. Data reported within Central Eastern European drainage systems were taken only. Altogether 52 relevant 'European' articles were collected encompassing metadata on length and age (wild and captive), frequently encountered length (in rivers), condition factor (wild and captive), growth and thermal conditions of growth (wild and captive), length increment (wild and captive), natural diet composition (with ontogenic shift), body composition (wild and captive), different feeding treatments in captivity and their performance (growth, feed utilization, yield, condition). Altogether a database of 1581 raw data was compiled (available on request).

Our experiments' observed TGC, length increment, density, and yield was compared with meta-analyzed TGC from published growth trial results; segregated growth phase-age wise (i.e. exponential 0+ age, and stabilized ≈1+). Meta-data from 8 published growth trials were used for a retrospective evaluation of our obtained results [15-22]. Length at age, length-weight relationship, condition factor, length increment, thermal conditions in the present growth trial was compared with the wild estimates from European rivers. Metadata from 18 observations in the wild were used [21, 23-38]. Additionally, metadata on some hatchery-raised and stocked barbels in European rivers were used [22, 24, 39, 40].

A 'riverine stocking suitability' of barbels obtained from our growth trials, employing smart nutrition decisions, was empirically assessed. Mean body length obtained from optimum dietary treatments in growth trial 1 (age late 0+/ 8 months) or validation growth trial 2 (age early 1+/ 13 months) were compared with the body length of juvenile to sub-adult barbels (=25 cm; [25] encountered in European rivers. Metadata on the frequent or representative body length of barbels below 25 cm length observed in European rivers were taken from 8 wild estimates [24, 28, 30, 36, 41-44].

3. Results

Optimum nutrition for achieving steepest growth trajectory

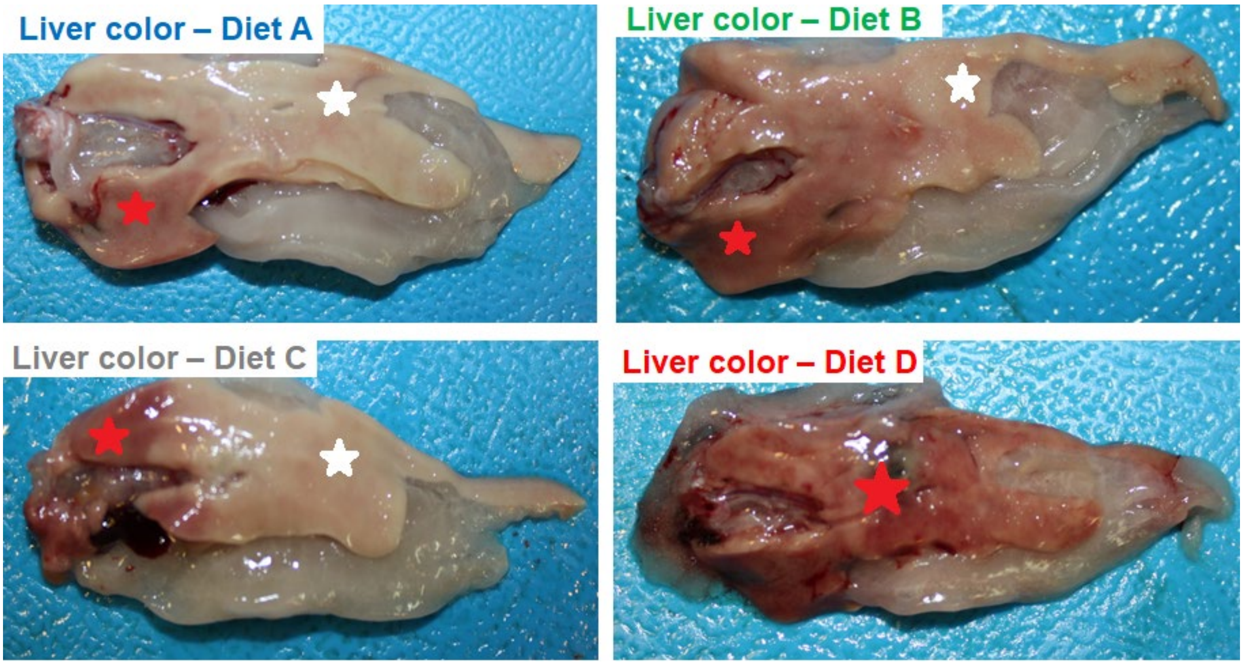

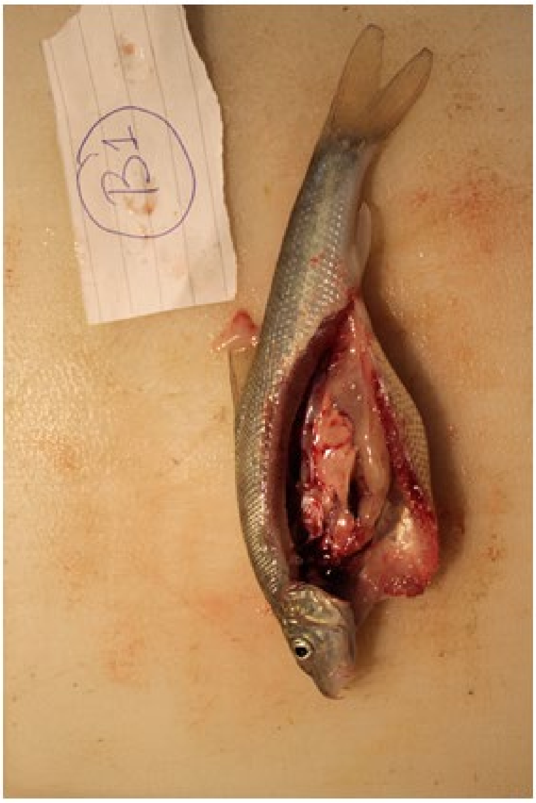
B1
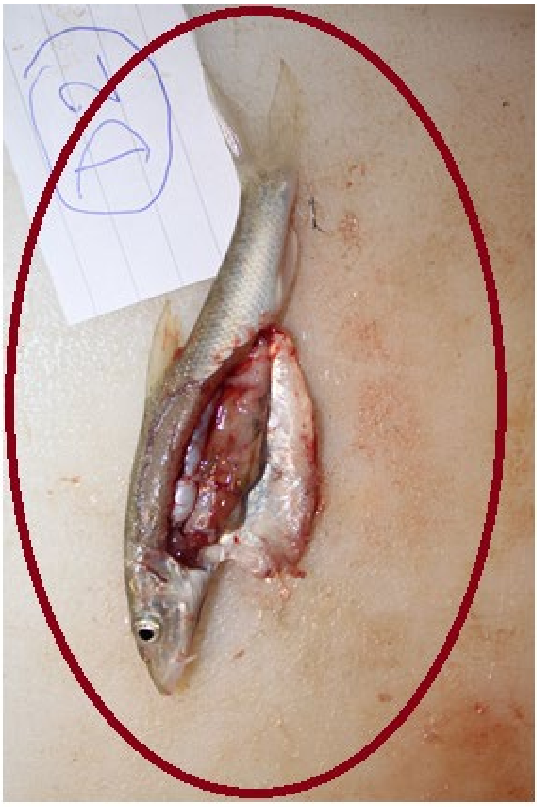
D2
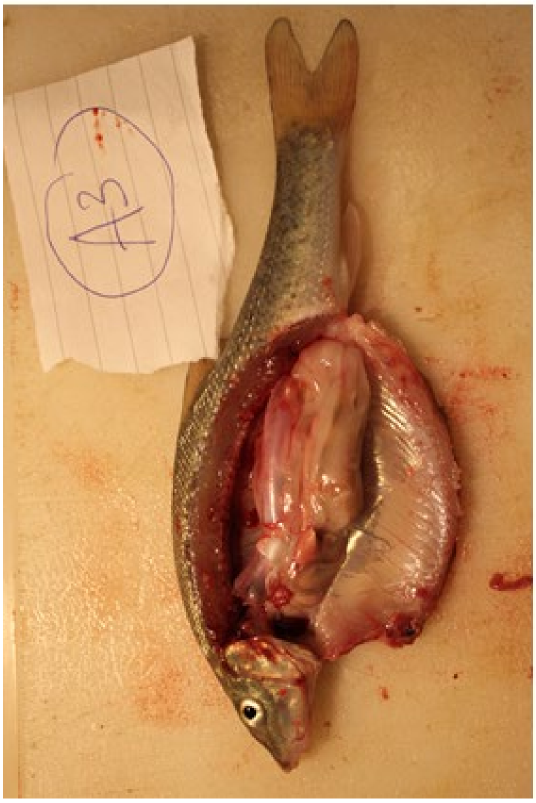
A3
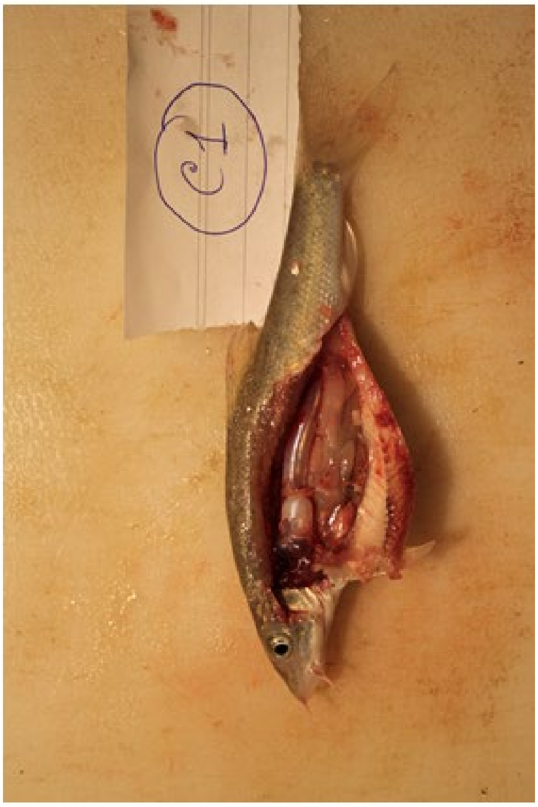
C1

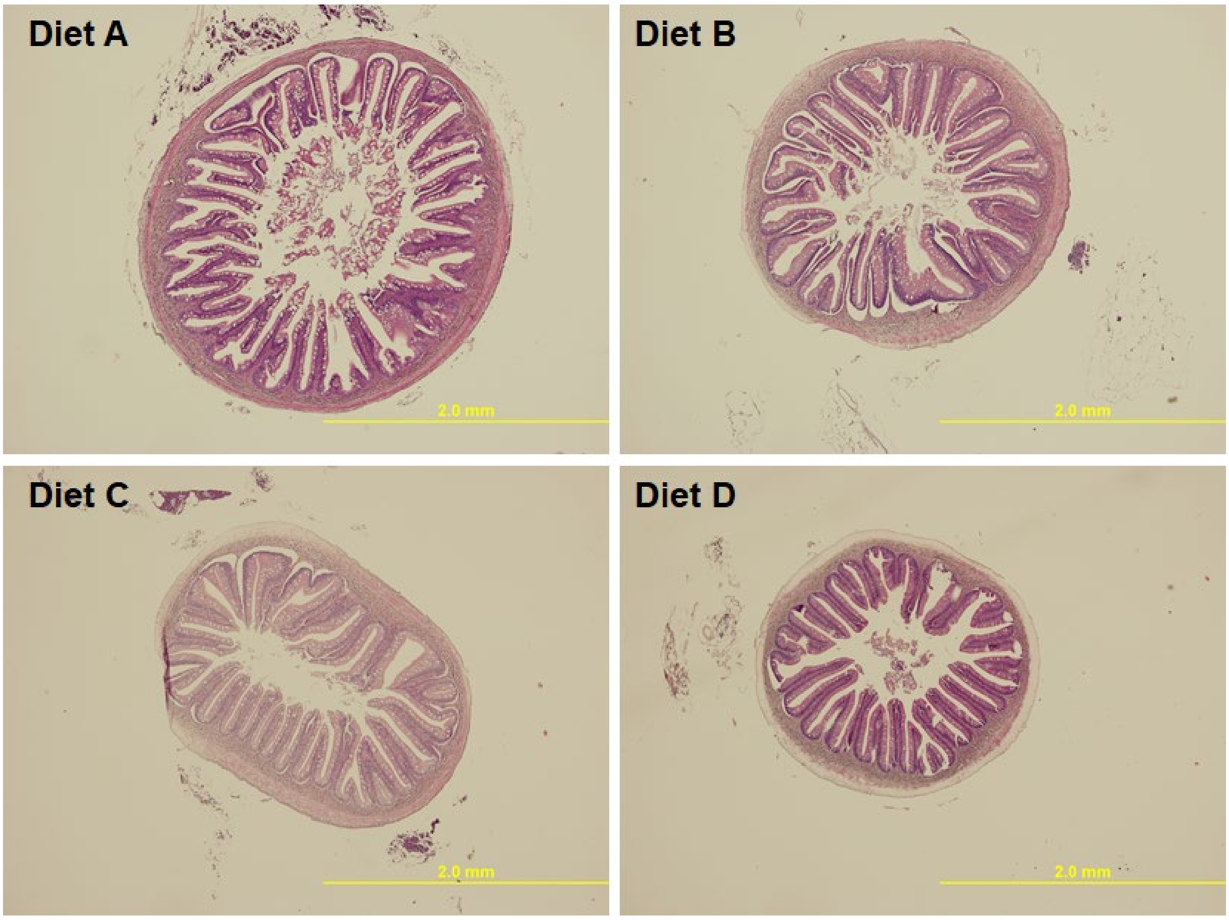
Diet A
2.0 mm
Diet B
2.0 mm
Diet C
2.0 mm
Diet D
2.0 mm

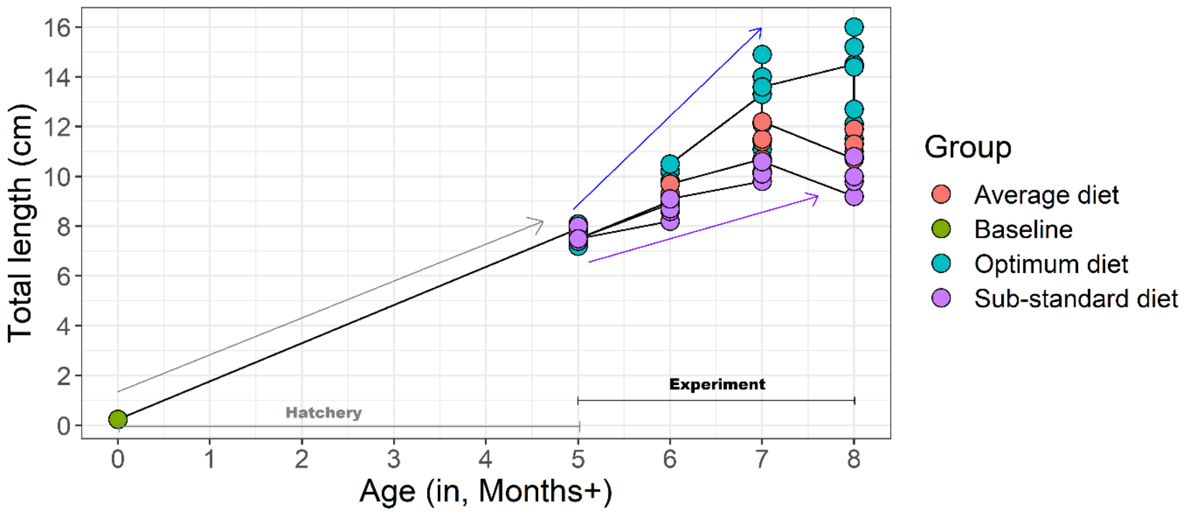
Total length (cm)
Age (in, Months+)
Hatchery
Experiment
Group
Average diet
Baseline
Optimum diet
Sub-standard diet

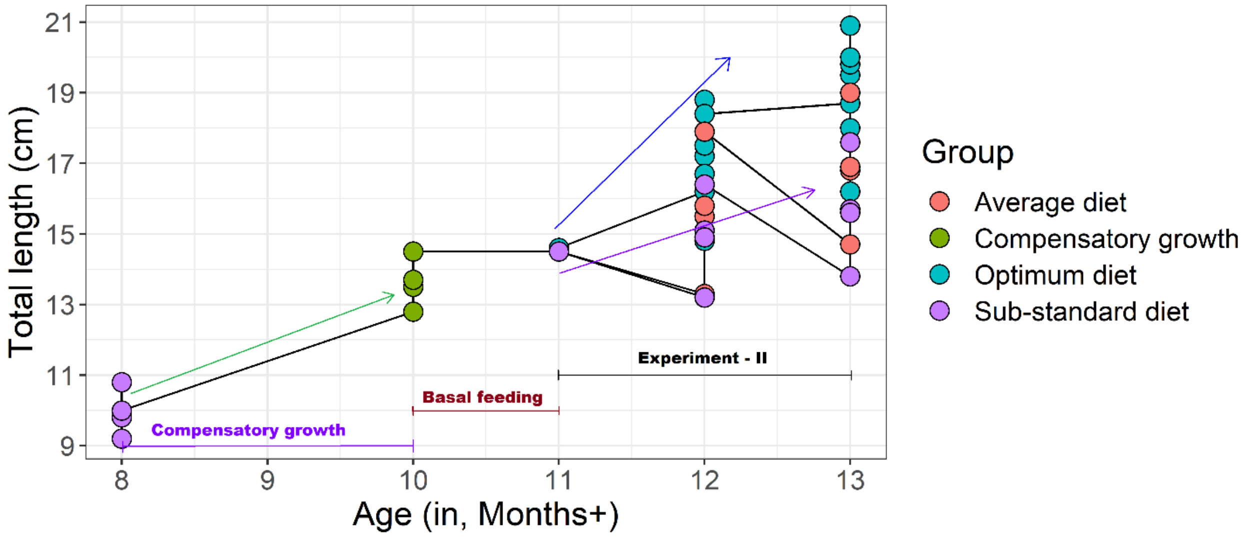
Total length (cm)
Age (in, Months+)
Compensatory growth
Basal feeding
Experiment - II
Group
Average diet
Compensatory growth
Optimum diet
Sub-standard diet

3.3. Understanding nutritional physiology and conditions for achieving maximum growth

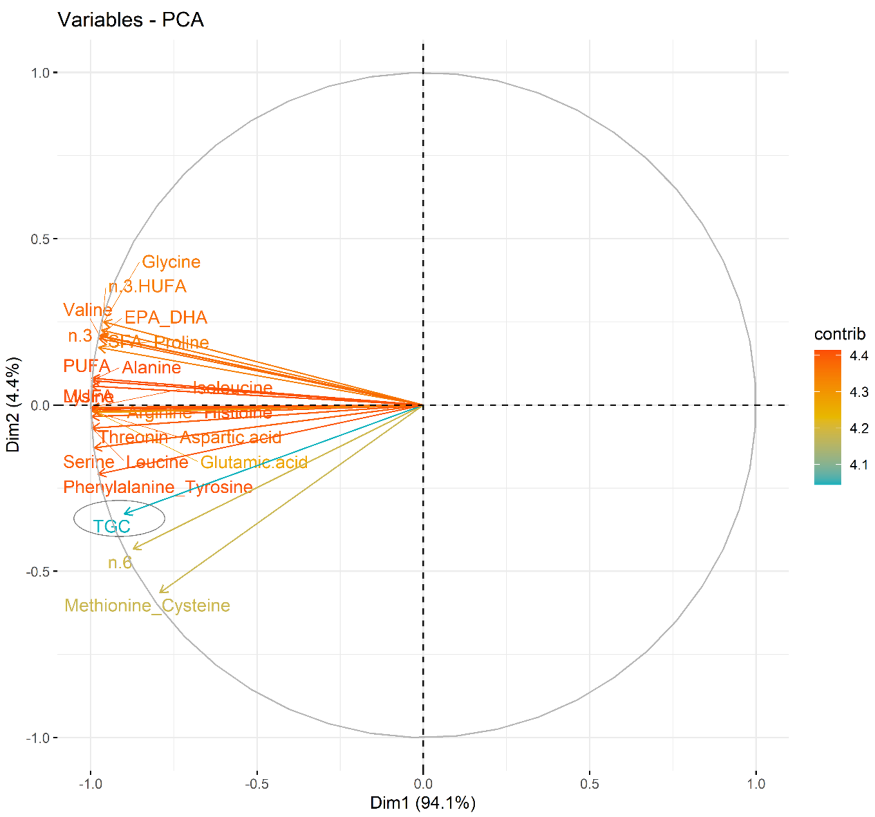

Figure S5: Deep PCA among amino acids, fatty acids, and growth (thermal growth coefficient, TGC). Owing to our strategic diet selection, multi-collinearity exists among nutrients and they cluster around TGC indicating positive correlation with growth, which was quite obvious.

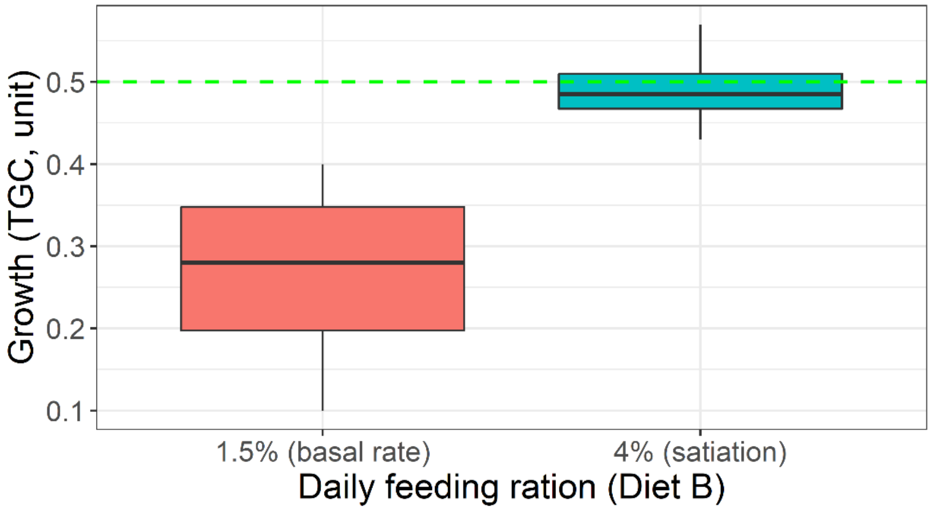

4. SUPPLEMENTARY DISCUSSIONS

Bigger and faster is better for riverine stocking success

Besides habitat restoration [45, 46] stocking has been used to increase or restore depleted fish populations [47] and in some cases has proven particularly successful [48-50]. Of course, there have been cases where the effect of stocking was variable and/or unclear [51-53]. Size-at-release is a vital parameter to consider for river stocking success[50, 54-63]. The logically intuitive hypothesis that larger or faster-growing members of a cohort gain a survival advantage over smaller conspecifics via enhanced resistance to starvation, decreased vulnerability to predators, and better tolerance of environmental extremes is referred to as the 'bigger is better' hypothesis [63]. The results generally support the "bigger is better" hypothesis [60, 63] when it comes to size-selective-mortality or survivability. Significant size-selective mortality of juveniles in the wild happens through overwintering mortality (for temperate species), starvation or intolerance of physical extremes by smaller members of the young-of-the-year (YOY) cohort, and predation, with smaller fish more susceptible to successful capture by predators [62-64].[62] showed that bigger fish perform better than smaller fish at higher water velocities; however, their performance can decrease significantly at shallow water depths. Although the response is species-specific, smaller-sized fish have an increased risk of fatigue and shorter endurance times than larger-sized fish swimming at the same water velocities and depths due to their higher metabolic demands [62].

Releases of artificially reared individuals are usual practices for sustaining populations of native species that need conservation. They are often released locally in river stretches, creating a situation of high prey abundance for predators [64]. Thus, predators heavily prey on released individuals, and predation is thought to cause decreased practical efficiency [65, 66]. To cite a few examples, evidence obtained by American salmonid hatcheries on the Pacific Coast indicates that survival of juvenile coho (*Oncorhynchus kisutch*) and chinook (*O. tshawytscha*) salmon can be influenced by the size of smolts at release (reviewed in [57]. [64] showed that piscivorous salmonids pursue schooling fry after riverine stocking, and small fry may be easily caught due to their slow speed in avoiding predators. [67] estimated that Atlantic salmon (*Salmo salar*) fry mortality in streams from predation by other salmonid predators like brook trout (*Salvelinus fontinalis*) and brown trout (*Salmo trutta*) ranged from 5% to 60% in the stocking programs in Massachusetts, USA. Predation on Chinook salmon fry (*O. tshawytscha*) by non-native fish in the San Joaquin River, USA, also decreased the efficiency of stocking programs [68]. A positive correlation between body size at release and recapture rate may reflect a crucial advantage of stocking large size-at-release fish [69-71], especially in avoiding size-selective predation by predators which are limited by their mouth gape size [59]. Predators like pike, common in European drainage systems, have also shown to be size-selective predators preferring small prey sizes (reviewed in [59]. Predation on relatively small, released individuals may decrease the practical efficiency of the population enhancement if the smaller individuals are more vulnerable to predation than larger ones. Thus, a bigger size at release could provide them with refuge from predation [59, 64] than focusing on releasing abundant numbers of small fry that may be inefficient (case study; [51].

However, bigger size-at-release also needs to be achieved in the right way, *i.e.,* uncompromised behavior (naivety to predation, foraging), uncompromised physique (morphology, physiology, fitness), and within economic compromises (logistics, housing, and feeding costs) is a complex task to accomplish (see, introduction). It basically means achieving a very steep growth trajectory within the shortest possible time (captivity). Hatchery-reared fish are originally inexperienced in dealing with predators and maybe an easy catch for the experienced piscivores [72]. They are also inexperienced in occupying feeding niches (habitat) from wild conspecifics or heterospecifics, which might displace

them easily [35]. Hence, the longer the time spent in captivity, the greater is the risk of behavioral deficits and predation or starvation mortality in the wild [58, 73]. To cite some examples in this regard, captive rearing is expected to impart some domestication effects on the fish [74, 75], such as altered morphology [76]; may be linked to nutritional deficiency), changes in foraging behavior [61, 77-79], and naivety to predators [79, 80]. [79] found that hatchery-reared Florida Bass (*Micropterus salmoides*) had significantly different movement, habitat selection, and higher mortality rates due to predation within the first 7 days of stocking. The abovementioned scientific evidence forms the basis of our 'bigger and faster is better' hypotheses (in partial modification of [63] in the context of riverine stocking.

Pre-release conditioning measures

While habitat restoration is a topic in its entirety, we comment on some 'pre-release conditioning measures' that could be applied to superior cohorts raised by smart, optimum nutrition. For example, depriving them of energy-dense artificial dry feeds, training them to feed on live insects or larvae [58, 81], or keeping the hatchery fish in pens or predator-free cages [80, 82, 83]. Adding habitat complexity (e.g., maze, refuge; non-reversing mirrors), altering water-flow velocities, supplementing diets with natural live foods, reducing rearing densities to more wild-like, exposure to predator and/or injured conspecifics exposure may reduce environmentally induced differences between cultured and wild fish [84-87]. Trippel et al. [88] found that exposing pellet-reared Florida Bass to natural prey, predators, and habitat for ten days in earthen ponds increased their post-stocking survivability. *In-situ* conditioning of hatchery fish in net pens might be another option for achieving higher survivability [89]. Off-season breeding [90] and rearing to advanced sizes [91], present study) under smart feeding of optimum diets (present study), combined with different conditioning measures mentioned above, can be potential solutions.

References

1. Roy, Koushik, Jaroslav Vrba, Sadasivam J Kaushik, and Jan Mraz. "Nutrient Footprint and Ecosystem Services of Carp Production in European Fishponds in Contrast to Eu Crop and Livestock Sectors." *Journal of Cleaner Production* 270 (2020): 122268.
2. Roy, Koushik, Jaroslav Vrba, Sadasivam J Kaushik, and Jan Mraz. "Feed-Based Common Carp Farming and Eutrophication: Is There a Reason for Concern?" *Reviews in Aquaculture* 12, no. 3 (2020): 1736-58.
3. Heinitz, MC, A Lemme, and Carsten Schulz. "Measurement of Digestibility in Agastric Fish Based on Stripping Method–Apparent Nutrient, Energy and Amino Acid Digestibilities of Common Feed Ingredients for Carp Diets (Cyprinus Carpio)." *Aquaculture Nutrition* 22, no. 5 (2016): 1065-78.
4. Yamamoto, Takeshi, Atsushi Akimoto, Sohtaroh Kishi, Tatsuya Unuma, and Toshio Akiyama. "Apparent and True Availabilities of Amino Acids from Several Protein Sources for Fingerling Rainbow Trout, Common Carp, and Red Sea Bream." *Fisheries science* 64, no. 3 (1998): 448-58.
5. Bláhová, Zuzana, Thomas Nelson Harvey, Martin Pšenička, and Jan Mráz. "Assessment of Fatty Acid Desaturase (Fads2) Structure-Function Properties in Fish in the Context of Environmental Adaptations and as a Target for Genetic Engineering." *Biomolecules* 10, no. 2 (2020): 206.
6. Turchini, Giovanni M, Bente E Torstensen, and Wing-Keong Ng. "Fish Oil Replacement in Finfish Nutrition." *Reviews in Aquaculture* 1, no. 1 (2009): 10-57.
7. NRC, National Research Council. *Nutrient Requirements of Fish and Shrimp*: National academies press, 2011.
8. Hardy, Ronald W, and Frederick T Barrows. "Diet Formulation and Manufacture." In *Fish Nutrition*, 505-600: Elsevier, 2003.
9. Froese, Rainer. "Cube Law, Condition Factor and Weight–Length Relationships: History, Meta-Analysis and Recommendations." *Journal of Applied Ichthyology* 22, no. 4 (2006): 241-53.
10. Noori, Nazanin, Kamyar Kalantar-Zadeh, Csaba P Kovesdy, Rachelle Bross, Debbie Benner, and Joel D Kopple. "Association of Dietary Phosphorus Intake and Phosphorus to Protein Ratio with Mortality in Hemodialysis Patients." *Clinical Journal of the American Society of Nephrology* 5, no. 4 (2010): 683-92.
11. Vize, Peter D. "A Homeric View of Kidney Evolution: A Reprint of Hw Smith's Classic Essay with a New Introduction." *The Anatomical Record Part A: Discoveries in Molecular, Cellular, and Evolutionary Biology: An Official Publication of the American Association of Anatomists* 277, no. 2 (2004): 344-54.
12. Sugiura, Shozo H, Faye M Dong, and Ronald W Hardy. "A New Approach to Estimating the Minimum Dietary Requirement of Phosphorus for Large Rainbow Trout Based on Nonfecal Excretions of Phosphorus and Nitrogen." *The Journal of Nutrition* 130, no. 4 (2000): 865-72.

13. Lunda, Roman, Koushik Roy, Petr Dvorak, Antonin Kouba, and Jan Mraz. "Recycling Biofloc Waste as Novel Protein Source for Crayfish with Special Reference to Crayfish Nutritional Standards and Growth Trajectory." *Scientific Reports* 10, no. 1 (2020): 1-10.
14. Poncin, Pascal. "Effects of Different Photoperiods on the Reproduction of the Barbel, Barbus Barbus (L.), Reared at Constant Temperature." *Journal of Fish Biology* 35, no. 3 (1989): 395-400.
15. Fiala, J, and P Spurny. "Intensive Rearing of Juvenile Barbel (Barbus Barbus) under Controlled Conditions." Paper presented at the [4. Czech Ichthyological Conference], Vodnany (Czech Republic), 10-12 May 2000 2000.
16. Kamiński, RafaŁ, Ewa Kamler, Jacek Wolnicki, Justyna Sikorska, and Jakub WaŁowski. "Condition, Growth and Food Conversion in Barbel, Barbus Barbus (L.) Juveniles under Different Temperature/Diet Combinations." *Journal of Thermal Biology* 35, no. 8 (2010): 422-27.
17. Myszkowski, L. "Compensatory Growth, Condition and Food Utilization in Barbel Barbus Barbus Juveniles Reared at Different Feeding Periodicities with a Dry Diet." *Journal of Fish Biology* 82, no. 1 (2013): 347-53.
18. Philippart, Jean-Claude, Ch Mélard, and Pascal Poncin. "Intensive Culture of the Common Barbel, Barbus Barbus (L.) for Restocking." (1989).
19. Policar, Tomáš, Pavel Kozák, Jitka Hamáčková, Andrea Lepičová, Jiří Musil, and Jan Kouřil. "Effects of Short-Time Artemia Spp. Feeding in Larvae and Different Rearing Environments in Juveniles of Common Barbel (Barbus Barbus) on Their Growth and Survival under Intensive Controlled Conditions." *Aquatic Living Resources* 20, no. 2 (2007): 175-83.
20. Policar, T, P Podhorec, V Stejskal, Pi Kozák, V Švinger, and SM Hadi Alavi. "Growth and Survival Rates, Puberty and Fecundity in Captive Common Barbel (Barbus Barbus L.) under Controlled Conditions." *Czech Journal of Animal Science* 56, no. 10 (2011): 433-42.
21. Kouba, Antonín, Josef Velíšek, Alžběta Stará, Jiří Masojídek, and Pavel Kozák. "Supplementation with Sodium Selenite and Selenium-Enriched Microalgae Biomass Show Varying Effects on Blood Enzymes Activities, Antioxidant Response, and Accumulation in Common Barbel (Barbus Barbus)." *BioMed research international* 2014 (2014).
22. Pegg, Josephine, and J Robert Britton. "Effects of Inter-and Intra-Specific Competition on the Growth Rates of Juvenile European Barbel Barbus Barbus Used in the Stock Enhancement of Uk Fisheries." *Fisheries Research* 112, no. 1-2 (2011): 8-12.
23. Baras, Etienne, and Jean-Claude Philippart. "Adaptive and Evolutionary Significance of a Reproductive Thermal Threshold in Barbus Barbus." *Journal of Fish Biology* 55, no. 2 (1999): 354-75.
24. Bašić, Tea, and J Robert Britton. "Characterizing the Trophic Niches of Stocked and Resident Cyprinid Fishes: Consistency in Partitioning over Time, Space and Body Sizes." *Ecology and evolution* 6, no. 14 (2016): 5093-104.
25. Benitez, Jean-Philippe, and Michaël Ovidio. "The Influence of Environmental Factors on the Upstream Movements of Rheophilic Cyprinids According to Their Position in a River Basin." *Ecology of Freshwater Fish* 27, no. 3 (2018): 660-71.
26. Bischoff, Antje, and Jörg Freyhof. "Seasonal Shifts in Day-Time Resource Use of 0+ Barbel, Barbus Barbus." *Environmental Biology of Fishes* 56, no. 1 (1999): 199-212.
27. Carosi, A, L Ghetti, G La Porta, and M Lorenzoni. "Ecological Effects of the European Barbel Barbus Barbus (L., 1758)(Cyprinidae) Invasion on Native Barbel Populations in the Tiber River Basin (Italy)." *The European Zoological Journal* 84, no. 1 (2017): 420-35.
28. Djikanovic, V, Z Gacic, and P Cakic. "Endohelminth Fauna of Barbel Barbus Barbus(L. 1758) in the Serbian Section of the Danube River, with Dominance of Acanthocephalan Pomphorhynchus Laeavis." *Bulletin of the European Association of Fish Pathologists* 30, no. 6 (2010): 229-36.
29. Grund, Stefanie, Steffen Keiter, Melanie Böttcher, Nadja Seitz, Karl Wurm, Werner Manz, Henner Hollert, and Thomas Braunbeck. "Assessment of Fish Health Status in the Upper Danube River by Investigation of Ultrastructural Alterations in the Liver of Barbel Barbus Barbus." *Diseases of aquatic organisms* 88, no. 3 (2010): 235-48.
30. Hunt, PC, and JW Jones. "A Population Study of Barbus Barbus L. In the River Severn, England: Iii. Growth." *Journal of Fish Biology* 7, no. 3 (1975): 361-76.
31. Morina, Arian, Filis Morina, Vesna Djikanović, Sladjana Spasić, Jasmina Krpo-Ćetković, Bojan Kostić, and Mirjana Lenhardt. "Common Barbel (Barbus Barbus) as a Bioindicator of Surface River Sediment Pollution with Cu and Zn in Three Rivers of the Danube River Basin in Serbia." *Environmental Science and Pollution Research* 23, no. 7 (2016): 6723-34.
32. Prokes, Miroslav, Pavol Sovcik, Milan Penaz, Vlastimil Barus, Petr Spurny, and Lorenzo Vilizzi. "Growth of Barbel, Barbus Barbus, in the River Jihlava Following Major Habitat Alteration and Estimated by Two Methods." *FOLIA ZOOLOGICA-PRAHA-* 55, no. 1 (2006): 86.
33. Przybylski, Mirosław, A Boron, and Andrzej Kruk. "Growth of Barbel, Barbus Barbus (L.) in the Upper Warta River, Odra River System." *Ecohydrology & Hydrobiology* 2 (2004): 183-90.
34. Roberts, Catherine Gutmann, and J Robert Britton. "Spawning Strategies in Cypriniform Fishes in a Lowland River Invaded by Non-Indigenous European Barbel Barbus Barbus." *Hydrobiologia* 847, no. 19 (2020): 4031-47.
35. Taylor, AAL, JR Britton, and IG Cowx. "Does the Stock Density of Stillwater Catch and Release Fisheries Affect the Growth Performance of Introduced Cultured Barbel?" *Journal of Fish Biology* 65 (2004): 308-13.
36. Trigo, Fatima Amat, Catherine Gutmann Roberts, and John Robert Britton. "Spatial Variability in the Growth of Invasive European Barbel Barbus Barbus in the River Severn Basin, Revealed Using Anglers as Citizen Scientists." *Knowledge & Management of Aquatic Ecosystems*, no. 418 (2017): 17.
37. Vilizzi, L, GH Copp, and JR Britton. "Age and Growth of European Barbel Barbus Barbus (Cyprinidae) in the Small, Mesotrophic River Lee and Relative to Other Populations in England." *Knowledge and Management of Aquatic Ecosystems*, no. 409 (2013): 09.

38. Watkins, MS, S Doherty, and GH Copp. "Microhabitat Use by 0+ and Older Fishes in a Small English Chalk Stream." *Journal of Fish Biology* 50, no. 5 (1997): 1010-24.
39. Antognazza, Caterina Maria, Demetra Andreou, Serena Zaccara, and Robert J Britton. "Loss of Genetic Integrity and Biological Invasions Result from Stocking and Introductions of Barbus Barbus: Insights from Rivers in England." *Ecology and evolution* 6, no. 5 (2016): 1280-92.
40. Britton, JR, IG Cowx, and G Peirson. "Sources of Error in the Ageing of Stocked Cyprinids." *Fisheries Management and Ecology* 11, no. 6 (2004): 415-17.
41. Copp, GH, and TA Bennetts. "Short-Term Effects of Removing Riparian and Instream Cover on Barbel (Barbus Barbus) and Other Fish Populations in a Stretch of English Chalk Stream." *Folia Zoologica (Czech Republic)* (1996).
42. Penaz, Milan, Vlastimil Barus, Miroslav Prokes, and Miloslav Homolka. "Movements of Barbel, Barbus Barbus (Pisces: Cyprinidae)." *Folia Zoologica (Czech Republic)* (2002).
43. Prchalová, Marie, Lukás Vetesník, and Ondrej Slavík. "Migrations of Juvenile and Subadult Fish through a Fishpass During Late Summer and Fall." *Folia Zoologica* 55, no. 2 (2006): 162.
44. De Santis, Vanessa, Catherine Gutmann Roberts, and J Robert Britton. "Influences of Angler Subsidies on the Trophic Ecology of European Barbel Barbus Barbus." *Fisheries Research* 214 (2019): 35-44.
45. Geist, Juergen. "Seven Steps Towards Improving Freshwater Conservation." Wiley Online Library, 2015.
46. Geist, Juergen, and Stephen J Hawkins. "Habitat Recovery and Restoration in Aquatic Ecosystems: Current Progress and Future Challenges." *Aquatic Conservation: Marine and Freshwater Ecosystems* 26, no. 5 (2016): 942-62.
47. Garlock, TM, EV Camp, and K Lorenzen. "Using Fisheries Modeling to Assess Candidate Species for Marine Fisheries Enhancement." *Fisheries Research* 186 (2017): 460-67.
48. Bell, Johann D, Devin M Bartley, Kai Lorenzen, and Neil R Loneragan. "Restocking and Stock Enhancement of Coastal Fisheries: Potential, Problems and Progress." *Fisheries Research* 80, no. 1 (2006): 1-8.
49. Crook, David A, Damien J O'Mahony, Bronwyn M Gillanders, Andrew R Munro, Andrew C Sanger, Stephen Thurstan, and Lee J Baumgartner. "Contribution of Stocked Fish to Riverine Populations of Golden Perch (Macquaria Ambigua) in the Murray–Darling Basin, Australia." *Marine and Freshwater Research* 67, no. 10 (2015): 1401-09.
50. Mesing, Charles L, Richard L Cailteux, P Andrew Strickland, Eric A Long, and Mark W Rogers. "Stocking of Advanced-Fingerling Largemouth Bass to Supplement Year-Classes in Lake Talquin, Florida." *North American Journal of Fisheries Management* 28, no. 6 (2008): 1762-74.
51. García-Vega, Ana, Pedro M Leunda, José Ardaiz, and Francisco Javier Sanz-Ronda. "Effect of Restoration Measures in Atlantic Rivers: A 25-Year Overview of Sea and Riverine Brown Trout Populations in the River Bidasoa." *Fisheries Management and Ecology* 27, no. 6 (2020): 580-90.
52. Mueller, Melanie, Joachim Pander, and Juergen Geist. "Comprehensive Analysis of> 30 Years of Data on Stream Fish Population Trends and Conservation Status in Bavaria, Germany." *Biological Conservation* 226 (2018): 311-20.
53. Vehanen, T. "Factors Influencing the Yield of Brown Trout, Salmo Trutta M. Lacustris L., in Northern Finnish Lakes." *Fisheries Management and Ecology* 2, no. 2 (1995): 121-34.
54. Colvin, N Elizabeth, Christopher L Racey, and Steve E Lochmann. "Stocking Contribution and Growth of Largemouth Bass Stocked at 50 and 100 Mm into Backwaters of the Arkansas River." *North American Journal of Fisheries Management* 28, no. 2 (2008): 434-41.
55. Diana, Matthew J, and David H Wahl. "Long-Term Stocking Success of Largemouth Bass and the Relationship to Natural Populations." Paper presented at the American Fisheries Society Symposium 2008.
56. Hoxmeier, RJH, and DAVID H Wahl. "Evaluation of Supplemental Stocking of Largemouth Bass across Reservoirs: Effects of Predation, Prey Availability, and Natural Recruitment." Paper presented at the American Fisheries Society Symposium 2002.
57. Bilton, HT, DF Alderdice, and JT Schnute. "Influence of Time and Size at Release of Juvenile Coho Salmon (Oncorhynchus Kisutch) on Returns at Maturity." *Canadian Journal of Fisheries and Aquatic Sciences* 39, no. 3 (1982): 426-47.
58. Brown, Culum, and Rachel L Day. "The Future of Stock Enhancements: Lessons for Hatchery Practice from Conservation Biology." *Fish and Fisheries* 3, no. 2 (2002): 79-94.
59. Hyvärinen, Pekka, and Teppo Vehanen. "Effect of Brown Trout Body Size on Post-Stocking Survival and Pike Predation." *Ecology of Freshwater Fish* 13, no. 2 (2004): 77-84.
60. Lorenzen, Kai. "Allometry of Natural Mortality as a Basis for Assessing Optimal Release Size in Fish-Stocking Programmes." *Canadian Journal of Fisheries and Aquatic Sciences* 57, no. 12 (2000): 2374-81.
61. Porak, WF, WE Johnson, S Crawford, DJ Renfro, TR Schoeb, RB Stout, RA Krause, and RA DeMauro. "Factors Affecting Survival of Largemouth Bass Raised on Artificial Diets and Stocked into Florida Lakes." Paper presented at the American Fisheries Society Symposium 2002.
62. Shiau, Jenny, Jabin R Watson, Rebecca L Cramp, Matthew A Gordos, and Craig E Franklin. "Interactions between Water Depth, Velocity and Body Size on Fish Swimming Performance: Implications for Culvert Hydrodynamics." *Ecological Engineering* 156 (2020): 105987.
63. Sogard, Susan M. "Size-Selective Mortality in the Juvenile Stage of Teleost Fishes: A Review." *Bulletin of marine science* 60, no. 3 (1997): 1129-57.
64. Hasegawa, Koh, Kentaro Honda, Taku Yoshiyama, Kengo Suzuki, and Sho Fukui. "Small Biased Body Size of Salmon Fry Preyed Upon by Piscivorous Fish in Riverine and Marine Habitats." *Canadian Journal of Fisheries and Aquatic Sciences* 78, no. 5 (2021): 631-38.
65. Moseby, KE, JL Read, DC Paton, P Copley, BM Hill, and HA Crisp. "Predation Determines the Outcome of 10 Reintroduction Attempts in Arid South Australia." *Biological Conservation* 144, no. 12 (2011): 2863-72.

66. Östman, Örjan, Maria K Boström, Ulf Bergström, Jan Andersson, and Sven-Gunnar Lunneryd. "Estimating Competition between Wildlife and Humans–a Case of Cormorants and Coastal Fisheries in the Baltic Sea." *Plos One* 8, no. 12 (2013): e83763.
67. Henderson, J Nathan, and Benjamin H Letcher. "Predation on Stocked Atlantic Salmon (Salmo Salar) Fry." *Canadian Journal of Fisheries and Aquatic Sciences* 60, no. 1 (2003): 32-42.
68. Michel, Cyril J, Joseph M Smith, Brendan M Lehman, Nicholas J Demetras, David D Huff, Patricia L Brandes, Joshua A Israel, Thomas P Quinn, and Sean A Hayes. "Limitations of Active Removal to Manage Predatory Fish Populations." *North American Journal of Fisheries Management* 40, no. 1 (2020): 3-16.
69. Salminen, M, S Kuikka, and E Erkamo. "Annual Variability in Survival of Sea-Ranched Baltic Salmon, Salmo Salar L: Significance of Smolt Size and Marine Conditions." *Fisheries Management and Ecology* 2, no. 3 (1995): 171-84.
70. Skurdal, J, O Hegge, and T Hesthagen. "Exploitation Rate, Survival and Movements of Brown Trout (Salmo Trutta L.) Stocked at Takeable Size in the Regulated Rivers Laagen and Otta, Southern Norway." *Regulated rivers: Research & management* 3, no. 1 (1989): 247-53.
71. Tipping, Jack M. "Effect of Smolt Length at Release on Adult Returns of Hatchery-Reared Winter Steelhead." *The Progressive fish-culturist* 59, no. 4 (1997): 310-11.
72. Olla, BL. "Behavioural Deficits in Hatchery-Reared Fish: Potential Effects on Survival Following Release." *Aquacult. Fish. Manag.* 25 (1994): 19-34.
73. Huntingford, Felicity A. "Implications of Domestication and Rearing Conditions for the Behaviour of Cultivated Fishes." *Journal of Fish Biology* 65 (2004): 122-42.
74. Grant, W Stewart, James Jasper, Dorte Bekkevold, and Milo Adkison. "Responsible Genetic Approach to Stock Restoration, Sea Ranching and Stock Enhancement of Marine Fishes and Invertebrates." *Reviews in Fish Biology and Fisheries* 27, no. 3 (2017): 615-49.
75. Lorenzen, Kai, Malcolm CM Beveridge, and Marc Mangel. "Cultured Fish: Integrative Biology and Management of Domestication and Interactions with Wild Fish." *Biological Reviews* 87, no. 3 (2012): 639-60.
76. Wintzer, AP, and PJ Motta. "Diet-Induced Phenotypic Plasticity in the Skull Morphology of Hatchery-Reared Florida Largemouth Bass, Micropterus Salmoides Floridanus." *Ecology of Freshwater Fish* 14, no. 4 (2005): 311-18.
77. Pouder, William F, Nicholas A Trippel, and Jason R Dotson. "Comparison of Mortality and Diet Composition of Pellet-Reared Advanced-Fingerling and Early-Cohort Age-0 Wild Largemouth Bass through 90 Days Poststocking at Lake Seminole, Florida." *North American Journal of Fisheries Management* 30, no. 5 (2010): 1270-79.
78. Rachels, Kyle T, Gordon R Taylor, Brandon M Baumhoer, Sagar Shrestha, and Steve E Lochmann. "Pellet-Reared Largemouth Bass Competitive Ability at Various Levels of Exposure to Live Forage." Paper presented at the Proceedings of the Annual Conference of the Southeast Association of Fish and Wildlife Agencies 2012.
79. Thompson, Brandon C, Wesley F Porak, Erin H Leone, and Micheal S Allen. "Using Radiotelemetry to Compare the Initial Behavior and Mortality of Hatchery-Reared and Wild Juvenile Florida Bass." *Transactions of the American Fisheries Society* 145, no. 2 (2016): 374-85.
80. Schlechte, J Warren, and David L Buckmeier. "A Pond Evaluation of Habituation as a Means to Reduce Initial Mortality Associated with Poststocking Predation of Hatchery-Reared Largemouth Bass." *North American Journal of Fisheries Management* 26, no. 1 (2006): 119-23.
81. Turek, Jan, Sabine Sampels, Sarvenaz KHALILI TILAMI, Daniel Červený, Jitka Kolářová, Tomáš Randák, Jan Mráz, Jan Másílko, Christoph Steinbach, and Viktoria Burkina. "Insects in the Feed of Rainbow Trout, Oncorhynchus Mykiss (Actinopterygii, Salmonidae): Effect on Growth, Fatty Acid Composition, and Sensory Attributes." *Acta Ichthyologica et Piscatoria* 50, no. 2 (2020).
82. Brown, C, and K Laland. "Social Learning and Life Skills Training for Hatchery Reared Fish." *Journal of Fish Biology* 59, no. 3 (2001): 471-93.
83. Brown, Grant E, and R Jan F Smith. "Acquired Predator Recognition in Juvenile Rainbow Trout (Oncorhynchus Mykiss): Conditioning Hatchery-Reared Fish to Recognize Chemical Cues of a Predator." *Canadian Journal of Fisheries and Aquatic Sciences* 55, no. 3 (1998): 611-17.
84. Berejikian, Barry A, E Paul Tezak, Thomas A Flagg, Anita L LaRae, Eric Kummerow, and Conrad VW Mahnken. "Social Dominance, Growth, and Habitat Use of Age-0 Steelhead (Oncorhynchus Mykiss) Grown in Enriched and Conventional Hatchery Rearing Environments." *Canadian Journal of Fisheries and Aquatic Sciences* 57, no. 3 (2000): 628-36.
85. Flagg, Thomas Alvin, Colin E Nash, Brian R Beckman, Barry A Berejikian, John Colt, Walton W Dickhoff, William T Fairgrieve, Robert N Iwamoto, Donald A Larsen, and CVW Mahnken. "A Conceptual Framework for Conservation Hatchery Strategies for Pacific Salmonids." (1999).
86. Hubená, Pavla, Pavel Horký, and Ondřej Slavík. "Performance of Cyprinids in Non-Reversing Mirrors Versus Regular Mirrors in Tests of Aggressiveness." *Journal of Ethology* 39, no. 1 (2021): 97-105.
87. Einum, Sigurd, and IA Fleming. "Implications of Stocking: Ecological Interactions between Wild and Released Salmonids." *Nordic Journal of Freshwater Research* 75 (2001): 56-70.
88. Trippel, Nicholas A., Wesley F. Porak, and Erin H. Leone. "Poststocking Survival of Conditioned and Pond-Reared Compared to Indoor Pellet-Reared Advanced Fingerling Florida Bass." *North American Journal of Fisheries Management* 38, no. 5 (2018): 1039-49.
89. Brennan, Nathan P, Meaghan C Darcy, and Kenneth M Leber. "Predator-Free Enclosures Improve Post-Release Survival of Stocked Common Snook." *Journal of Experimental Marine Biology and Ecology* 335, no. 2 (2006): 302-11.

90. Matthews, Michael D, and Richard B Stout. "Out-of-Season Spawning Method for Florida Largemouth Bass to Produce Advanced-Sized Fingerlings by Early Spring." *North American Journal of Aquaculture* 75, no. 4 (2013): 524-31.
91. Thompson, Brandon C, Wesley F Porak, William F Pouder, and Edward V Camp. "Survival of Advanced-Fingerlings of Florida Largemouth Bass Stocked in Small Florida Lakes." *North American Journal of Fisheries Management* 40, no. 6 (2020): 1532-44.
92. Alavi, Sayyed Mohammad Hadi, Martin Pšenička, Tomáš Policar, Marek Rodina, Jitka Hamáčková, Pavel Kozák, and Otomar Linhart. "Sperm Quality in Male Barbus Barbus L. Fed Different Diets During the Spawning Season." *Fish Physiology and Biochemistry* 35, no. 4 (2009): 683-93.
93. Policar, T, P Podhorec, V Stejskal, J Hamackova, and SMH Alavi. "Fertilization and Hatching Rates and Larval Performance in Captive Common Barbel (Barbus Barbus L.) Throughout the Spawning Season." *Journal of Applied Ichthyology* 26, no. 5 (2010): 812-15.
94. Curtean-Bănăduc, Angela, Alexandru Burcea, Claudia-Maria Mihuţ, Vidar Berg, Jan Ludvig Lyche, and Doru Bănăduc. "Bioaccumulation of Persistent Organic Pollutants in the Gonads of Barbus Barbus (Linnaeus, 1758)." *Ecotoxicology and Environmental Safety* 201 (2020): 110852.

Supplementary Appendix –SI: Natural food composition of some rheophilic fish (cyprinids) from the Danube region. Share of food items in diet spectrum is mapped by color scale, green cells indicate 'primary' food item. Data most abundant for barbel and asp. Insufficient data on nase and vimba bream.

Rheophilic cyprinid	Food items						Source
	Forage fish	Macrozoobenthos	Zooplankton	Filamentous algae	Bivalves	Ballast*	
Asp	1.00						Krpo-Ćetković et al. 2010
Asp	0.98		0.02				Vasek et al. 2018
Asp	0.95	0.05					Vasek et al. 2018
Asp	0.20	0.70	0.10				Vasek et al. 2018
Asp	0.65	0.25	0.10				Vasek et al. 2018
Asp	0.81	0.19					Adamek et al. 2019
Barbel		0.95				0.05	Bischoff and Freyhoff 1999
Barbel		0.98				0.02	Senk and Aganovic 1968
Barbel		0.82		0.11		0.07	Adamek and Obrdlik 1977
Barbel		0.43		0.49		0.08	Adamek and Obrdlik 1977
Barbel		0.67		0.18		0.15	Adamek and Obrdlik 1977
Barbel		0.54		0.40		0.06	Losos et al. 1980
Barbel		0.99				0.01	Lenhardt et al. 1996
Barbel		0.80		0.10		0.10	Filipovic and Jankovic 1978
Barbel		0.38		0.60		0.02	Chergou et al. 2002
Barbel		1.00					Szito and Gyore 1996
Nase		0.57	0.32			0.11	Reckendorfer et al. 2001
Vimba bream				0.61	0.38	0.02	Okgerman et al. 2013
Vimba bream				0.48	0.52		Okgerman et al. 2013
*Ballast imply collateral non-nutritional entries in search of primary food (e.g. detritus, sand)							

Supplementary Appendix –SII: Proximate composition of some daily food items expected in the habitats of rheophilic fish. Most values (except insect meal, commercial product, country of origin: Germany) are of European freshwater origin and expressed in 100% dry matter basis.

Dietary item	Protein %	Lipid %	P %	Fibre %	Ash %	Carbohydrates %	Source
Chironomid larvae	56.8	4.4	0.99	4.78	21.2	18.48	Roy et al. 2021
Insect meal	58.8	13.3	1.2	13.3	5	12.2	IAFFD 2020
Zooplankton	66	8.5	1.3	8	8.5	8.9	Roy et al. 2021
Small fish	65.7	11.5	3.4	–	18.2	4.7	Schreckenbach et al. 2021
Bivalves	68.7	4.1	0.85	–	19.1	8.1	Stańczykowska 1984, Sicuro et al. 2010
Filamentous algae	14.5	0.8	0.23	15.6	39.2	30.0	Planas et al. 1996, Messyasz et al. 2015

Supplementary Appendix – SIII: List of barbel metadata sources used in the main text figures

Main text figure	Data sources
**Figure 2.** Length increment in captive (feeding regimen wise) versus wild *Barbus barbus*.	1. **Smart feeding-optimum diet** = diets A, B.<br>2. **Feeding-status quo**= diets C, D + some previous growth trials [15-20, 22].<br>3. **Wild**= European rivers metadata [23-25, 27, 30, 32, 34].
**Figure 3.** Body fitness (Fulton's condition factor) of wild versus captive (feeding regime wise) *Barbus barbus*.	1. **Smart feeding-optimum diet**= diets A and B.<br>2. **Feeding-status quo**= diets C, D + some previous growth trials [15-17, 19, 20, 92, 93].<br>3. **European rivers**= wild data [29, 31, 33, 94].
**Figure 4.** Growth trajectory or size-at-age of captive (under different feeding regimen) versus wild *Barbus barbus*.	1. **Smart feeding-optimum diet** = diets A, B.<br>2. **Feeding-status quo** = diets C, D + previous captive trials [15, 16, 20, 22, 24, 39].<br>3. **Wild** = European rivers metadata [23, 26, 27, 30, 32-38].
**Figure 5.** Size suitability of captive raised *Barbus barbus* under different feeding regimen for river stocking purposes.	1. **European rivers data**: [24, 28, 30, 36, 41-44].
